# Evidence that a naturally occurring single nucleotide polymorphism in the *RagC* gene of *Leishmania donovani* contributes to reduced virulence

**DOI:** 10.1371/journal.pntd.0009079

**Published:** 2021-02-23

**Authors:** Patrick Lypaczewski, Wen-Wei Zhang, Greg Matlashewski

**Affiliations:** Department of Microbiology and Immunology, McGill University, Montreal, Canada; Bernhard Nocht Institute for Tropical Medicine, Hamburg, Germany, GERMANY

## Abstract

Leishmaniasis is a widespread neglected tropical disease transmitted by infected sand flies resulting in either benign cutaneous infection or fatal visceral disease. *Leishmania donovani* is the principal species responsible for visceral leishmaniasis, yet an atypical *L*. *donovani* has become attenuated in several countries including Sri Lanka and causes cutaneous leishmaniasis. Previous studies have identified 91 genes altered in the atypical cutaneous *L*. *donovani* compared to typical visceral disease associated *L*. *donovani* including mutations in the *RagC* and *Raptor* genes that are part of the eukaryotic conserved TOR pathway and its upstream sensing pathway. In the present study, we investigate whether the RagC R231C mutation present in atypical cutaneous *L*. *donovani* introduced into the virulent *L*. *donovani* 1S2D chromosome by CRISPR gene editing could affect virulence for survival in visceral organs. Through bioinformatic analysis, we further investigated the presence of sensing pathway components upstream of TOR in *L*. *donovani* including RagC complexing proteins, RagA and Raptor. *L*. *donovani* 1S2D edited to express mutant RagC R231C were viable in promastigote but had reduced visceral parasitemia in infected BALB/c mice. The RagC R231C mutant retained the ability to interact with RagA and gene knockout experiments revealed that although the *RagA* gene was essential, the *RagC* gene was not essential under promastigote culture conditions but was essential for survival in the liver of experimentally infected mice. These results provide evidence that the TOR associated sensing pathway plays a prominent role in *L*. *donovani* visceral disease and the RagC R231C mutation contributed to the atypical pathology of cutaneous *L*. *donovani* in Sri Lanka.

## Author summary

The *Leishmania donovani* parasite is transmitted by infected sandflies and is the principal causative agent of visceral leishmaniasis, a neglected tropical disease with no available vaccine. The infection normally spreads to the liver, spleen and bone marrow and is fatal if untreated. In Sri Lanka however, *L*. *donovani* causes only skin pathology at the site of the sandfly bite and does not cause visceral disease. Previous genetic analysis of this atypical attenuated strain identified 91 gene mutations including single amino acid mutations in the *RagC* and *Raptor* genes that are part of the conserved eukaryotic TOR regulatory pathway and upstream sensing complexes. This study investigated whether members of the TOR and associated sensing pathways could be identified by bioinformatic analysis and the potential role a RagC mutation plays in the attenuation of *L*. *donovani* in Sri Lanka for causing visceral disease. This study provides novel insight into the role of the TOR and associated sensing pathways and the *RagC* single nucleotide polymorphism in *L*. *donovani* virulence.

## Introduction

Leishmaniasis is a neglected tropical disease present throughout developing countries and is caused by *Leishmania* protozoa parasites that are transmitted by infected sandflies [1]. There are over 20 species of *Leishmania* that infect humans of which most remain in the dermal layer of the skin at the site of the sand fly bite resulting in cutaneous leishmaniasis that usually self-cures [2]. The *Leishmania donovani* species however typically disseminates from the dermis to the liver, spleen and bone marrow resulting in visceral leishmaniasis with persistent fever, hepatosplenomegaly, internal bleeding, anemia and is fatal if not treated. Humans are the only known reservoir for *L*. *donovani* while virtually all cutaneous leishmaniasis causing species have animal reservoirs [3]. Parasite tropism plays a fundamental role in leishmaniasis virulence and pathogenesis because parasites that visceralize are deadly compared to parasites that remain in the skin that generally self-heal. Visceral leishmaniasis is the second most deadly vector borne parasitic infection after malaria. What controls disease pathology and tropism during infection remains poorly understood although this is largely dependent on the species of *Leishmania* [4].

*L*. *donovani* is endemic in the Indian subcontinent and East Africa where most cases of visceral leishmaniasis occur. Notably, *L*. *donovani* has recently evolved to also cause cutaneous leishmaniasis in some geographic locations [5]. For example, the number of cutaneous leishmaniasis cases in Sri Lanka continues to increase with over 3000 cases in 2018 while there is no transmission of visceral leishmaniasis [6]. Whole genome sequencing of a *L*. *donovani* cutaneous leishmaniasis strain in Sri Lanka identified a variety of single nucleotide polymorphisms (SNPs), indels and copy number variations including a non-conservative arginine to cysteine codon change at position 231 (R231C) in the *RagC* gene [7,8]. Determining the specific genetic changes responsible for the *L*. *donovani* conversion from a visceral to a cutaneous leishmaniasis strain would help define the molecular basis for disease tropisms and virulence associated with visceral leishmaniasis.

The life cycle of *Leishmania* includes the promastigote stage that replicates in the midgut of the sand fly vector and the amastigote stage that replicates in the phagolysosome of mammalian host macrophage cells. *Leishmania* must adapt to different environmental conditions including temperature, pH and nutrient availability to differentiate and proliferate in the different life cycle stages. In eukaryotes, the Mechanistic Target Of Rapamycin Complex 1 (mTORC1) within the TOR cell signaling pathway has multiple regulatory mechanisms controlling protein synthesis and autophagy [9]. Deregulation of the TOR pathway is implicated in the pathogenesis of various human diseases including cancer and immunological defects [10,11]. Three distinct TOR kinase genes have been identified in *Leishmania* [12] and it is largely unknown what other components of the TOR or TOR upstream sensing pathways are present in *Leishmania* and what role the mTORC1 complex plays in the *Leishmania* cell cycle, tropism and virulence. One of the *Leishmania* TOR kinases (TOR3) has been shown to be necessary for formation of the ancient acidocalcisome organelle associated with storage of calcium and the other 2 members (TOR1, TOR2) are essential for promastigote stage survival but their function is unknown [12].

**Fig 1:**
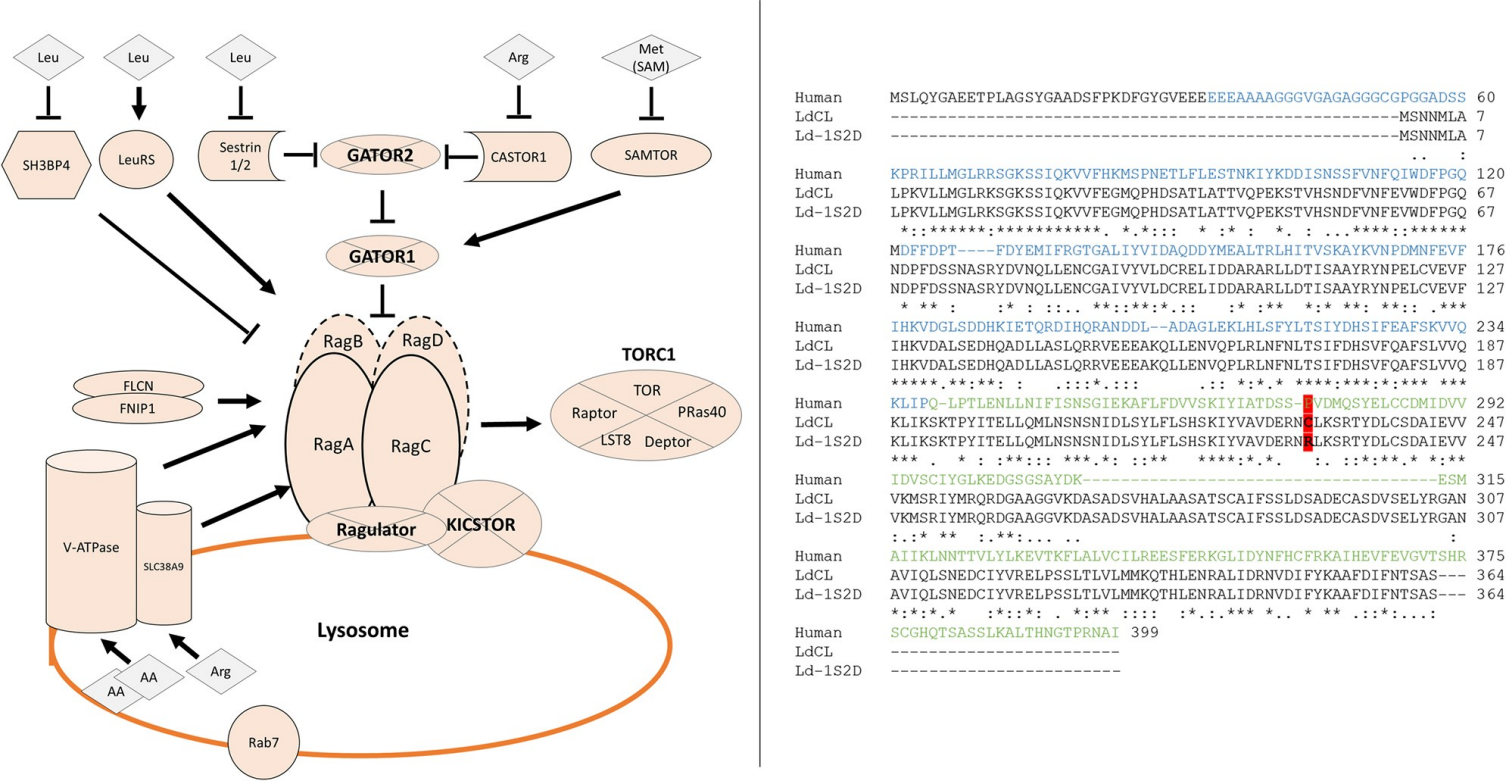
Comparison of the Rag pathway and sequence between humans and *Leishmania*. Left: The Rag sensing pathway upstream of mTORC1 signaling based on human studies. Cytosolic amino acid sensors responding to levels of Leucine, Arginine or Methionine are shown on top and inhibit Rag activation repressors or activate activators. Multi-protein complexes labeled in bold on segmented circles. Lysosomal amino acid sensors (vATP-ase and SLC38A9) and Rag heterodimer anchoring complex (Ragulator) shown on the lysosome. The Rag complex integrates signals from nutrient sensors in addition to growth factors (FLCN/FNIP1) resulting in modifications to its GTP/GDP bound state. Active state RagA-RagC bind Raptor effectively recruiting TORC1. *Note*: *The Rag protein dimer are overlapping on the human diagram as they perform the same function*. *This dimer consists either of RagA with RagC or of RagB with RagD*. Right: Multiple sequence alignment of human, wildtype and cutaneous *Leishmania* RagC proteins with domain structure labeled on the human RagC sequence with GTPase domain (blue) and C-terminal Roadblock domain (green). The R231C polymorphism identified in the cutaneous isolate is highlighted in red. Sequence identity is marked with (*), sequence similarity is marked with (:).

The mTORC1 associated pathway is well described in mammalian cells involving multiple regulatory proteins and protein complexes that respond to different kinds of nutrients and growth signals [13, see also Table 1 and Fig 1]. Accurate regulation of mTORC1 activity to control protein synthesis and autophagy is essential for cell growth, replication and survival [14]. Nutrients utilized by eukaryotic cells include carbohydrates, lipids, and amino acids. The Rag GTPases (Ras-related guanosine triphosphatases) have been identified as important mediators of amino acid signaling to mTORC1 in mammals (Fig 1). The Rag GTPases consist of RagA, RagB, RagC, and RagD. RagA and RagB with 90% sequence identity are functionally redundant. RagC and RagD are also functionally redundant and share 81% sequence identity. RagA or RagB bind to RagC or RagD to form a stable and functional heterodimeric complex. Like other GTP-binding proteins, the nucleotide loading state of the Rag GTPases regulates their function. Under amino acid-sufficient conditions, the RagA-RagC heterodimer localizes to the lysosomal surface and is transformed into the active form RagA^GTP^-RagC^GDP^ through the Ragulator complex [9,15]. The active RagA^GTP^-RagC^GDP^ then binds to Raptor resulting in the recruitment of mTORC1 to the lysosome [16,17]. Once mTORC1 is recruited to the lysosome, the TOR serine-threonine kinase is activated by Rheb [18] and phosphorylates its canonical substrates S6K1, 4EBP1, ULK1 and TFEB. Phosphorylation of S6K1 and 4EBP1 promotes protein synthesis and cell growth, while phosphorylation of ULK1 and TFEB inhibits autophagy.When amino acids are scarce, the Rag heterodimer is inactivated to form RagA^GDP^-RagC^GTP^ which detaches from Raptor resulting in dislocation of mTORC1 from lysosome and inactivation of mTORC1, thus suppresses further protein synthesis, and induces autophagy if necessary to maintain cell survival [9,18].

In the present study, we performed a bioinformatic analysis to identify potential members of the TOR and upstream sensing pathways in *L*. *donovani* to investigate how a naturally occurring mutation in the *RagC* (R231C) gene may contribute to reduced visceral pathogenesis. It was previously shown that introduction of a wildtype *RagC* gene in the cutaneous *L*. *donovani* strain by plasmid transfection resulted in an incremental but significant increased parasite survival in the visceral organs in experimentally infected mice providing evidence that the (R231C) mutation may have contributed to the attenuation for survival in visceral organs of this *L*. *donovani* strain in Sri Lanka [8]. To specifically investigate the role of the naturally occurring RagC R231C mutation in pathogenesis in the absence of other mutations present in the atypical cutaneous *L*. *donovani*, CRISPR gene editing was used to engineer the RagC R231C single amino acid mutant and a *RagC* gene disruption mutant in the wildtype virulent *L*. *donovani* 1S2D strain. We further undertook to disrupt the partner *RagA* gene and assessed whether the RagC/RagA interaction is conserved in *Leishmania* and if the RagC R231C point mutation affected the interaction with RagA. The results of this study provide evidence that the RagC R231C mutation in *L*. *donovani* plays a role in the attenuation of the atypical cutaneous *Leishmania* strain present in Sri Lanka and that several components of the TOR and upstream sensing pathways are conserved in *Leishmania*.

## Results

### Conservation of the amino acid sensing arm of the mTOR pathway

A complete *L*. *donovani* genome sequence from a cutaneous strain from Sri Lanka has recently closed over 2000 sequence gaps resulting in the identification of over 600 novel open reading frames [7]. Using sequence analysis (BLASTP) of this complete genome sequence, it was possible to identify conserved components of the TOR and upstream sensing pathways [9,14–18]. As shown in Figs 1 and S1, several components of this pathway are well conserved (BLASTP E-value < 0.001) between human cells and *L*. *donovani* (Table 1 and S1 Fig). Homologous components include sensors such as the v-ATPase or Leucyl-tRNA synthetase [19], signaling proteins including the RagA GTPase which is the binding partner for the RagC GTPase and cellular regulators such as the Raptor protein that acts as a bridge between the RagA/RagC heterodimer and the TOR kinase complex. However, several components present in mammalian eukaryotes are missing entirely or partially: The RagB/RagD complex, Ragulator complex, Folliculin complex, KICSTOR and CASTOR complexes were not found to any degree of homology in *Leishmania*. While the GATOR2 complex was present, only 1 member of the 3 protein GATOR1 complex was identified. Several of the TORC1 complex components were identified that had homology to the Raptor, TOR and LST8 proteins but not to the Deptor and PRas40 proteins (S1 Fig). These observations demonstrate that several components of the TOR and upstream sensing pathways are conserved between mammalian cells and *L*. *donovani* suggesting that a TOR signaling pathway is present in *Leishmania* but distinct from mammalian cells. Note also that mutations identified in the atypical cutaneous *L*. *donovani* strain are highlighted in the RagC and Raptor proteins [7](S1 Fig).

**Table 1:** List of *Leishmania donovani* proteins homologous to humans in the RagC axis of the TOR pathway. Proteins part of the RagC arm of the TOR pathway in humans and their parent complex names are listed along with UniProt accession numbers used for sequence retrieval. Matching *L*. *donovani* proteins resulting from a BLASTP search are listed when available along with their respective matching sequence length, percent identity, BLAST score and E-values. Homologues were marked as ABSENT when no protein was found. The 5^th^ column indicates the presence or absence of homologous proteins in *L*. *infantum (I)*, *L*. *major (Mj)*, *L*. *tropica (Tr)*, *L*. *mexicana (Mx) or L*. *Braziliensis (Br)*. When matching proteins were found in species other than *L*. *donovani* only, the BLASTP metrics are reflective of the top scoring hit in the indicated species.

Protein Name	Protein Complex	UniProt Accession	*L*. *donovani* Protein ID	Present in *Leishmania spp*	Coverage (Match/query)	Identity	BLAST score	E-value
CASTOR		Q8WTX7	ABSENT	None				
SEC13	**GATOR2**	P55735	LdCL_320005400	I, Mj, Tr, Mx, Br	310/322	40%	198	1.00E-58
WDR59		Q6PJI9	LdCL_300009300	I, Mj, Tr, Mx, Br	123/974	31%	70.1	7.00E-11
WDR24		Q96S15	LdCL_200015700	I, Mj, Tr, Mx, Br	69/790	43%	70.9	5.00E-11
SEH1L		Q96EE3	LdCL_320005400	I, Mj, Tr, Mx, Br	302/360	28%	99.8	1.00E-22
MIOS		Q9NXC5	LdCL_250018400	I, Mj, Tr, Mx, Br	82/875	40%	60.1	5.00E-08
NPRL2	**GATOR1**	Q8WTW4	ABSENT	None				
NPRL3		Q12980	ABSENT	None				
DEPDC		O75140	LdCL_070015500	I, Mj, Tr, Mx, Br	80/1603	34%	56.2	2.00E-06
Sestrin 1	**Sestrin**	Q9Y6P5	LdCL_180011700	I, Mj, Tr, Mx, Br	109/492	25%	49.7	3.00E-05
Sestrin 2		P58004	LdCL_180011700	I, Mj, Tr, Mx, Br	149/480	25%	54.3	1.00E-06
LeuRS		Q9P2J5	LdCL_130015300	I, Mj, Tr, Mx, Br	1089/1176	42%	843	0.00E+00
SH3BP4		Q9P0V3	ABSENT	None				
RagA		Q7L523	LdCL_130020300	I, Mj, Tr, Mx, Br	338/313	36%	218	2.00E-66
RagB		Q5VZM2	ABSENT	None				
RagC		Q9HB90	LdCL_360068800	I, Mj, Tr, Mx, Br	356/399	37%	231	3.00E-70
RagD		Q9NQL2	ABSENT	None				
LAMTOR1	**RAGULATOR**	Q6IAA8	ABSENT	None				
LAMTOR2		Q9Y2Q5	ABSENT	None				
LAMTOR3		Q9UHA4	ABSENT	None				
LAMTOR4		Q0VGL1	ABSENT	None				
LAMTOR5		O43504	ABSENT	None				
SLC38A9		Q8NBW4	LdCL_270011600	I, Mj, Tr, Mx, Br	161/562	22%	36.2	0.056
v-ATPase (subunit H)		Q9UI12	LdCL_210021400	I, Mj, Tr, Mx, Br	312/483	33%	153	1e-40
Rab7		P51149	LdCL_180014100	I, Mj, Tr, Mx, Br	227/207	52%	224	3.00E-72
KPTN	**KICSTOR**	Q9Y664	ABSENT	None				
SZT2		Q5T011	ABSENT	None				
C12Orf66		Q96MD2	ABSENT	Mj	136/445	26%	37	0.12
ITFG2		Q969R8	ABSENT	Mj	83/447	30%	34.3	0.99
Rheb		Q15382	LdCL_320027000	I, Mj, Tr, Mx, Br	156/184	37%	90.5	3.00E-21
FLCN		Q8NFG4	ABSENT	None				
FNIP1		Q8TF40	ABSENT	None				
SAMTOR		Q1RMZ1	LdCL_230020100	I, Mj, Tr, Mx, Br	88/405	35%	55.1	2.00E-07
Arf1		P84077	LdCL_310037200	I, Mj, Tr, Mx, Br	170/181	75%	281	9.00E-96
RuvB-like 1	**RuvB-like**	Q9Y265	LdCL_340041700	I, Mj, Tr, Mx, Br	455/456	70%	661	0.00E+00
RuvB-like 2		Q9Y230	LdCL_340032000	I, Mj, Tr, Mx, Br	449/463	70%	673	0.00E+00
Raptor	**mTORC1**	Q8N122	LdCL_250011400	I, Mj, Tr, Mx, Br	491/1335	37%	328	2.00E-91
Deptor		Q8TB45	ABSENT	None				
LST8		Q9BVC4	LdCL_100015100	I, Mj, Tr, Mx, Br	314/326	20%	70.1	2.00E-12
mTOR		P42345	LdCL_360073500	I, Mj, Tr, Mx, Br	2395/2549	29%	858	0.00E+00
			LdCL_340051700	I, Mj, Tr, Mx, Br	688/2549	41%	529	3.00E-152
			LdCL_340046900	I, Mj, Tr, Mx, Br	1211/2549	28%	478	8.00E-136
Pras40		Q96B36	ABSENT	None				

### Generation of the RagC R231C mutant and RagC disruption mutant from the virulent *L*. *donovani* 1S2D

**Fig 2:**
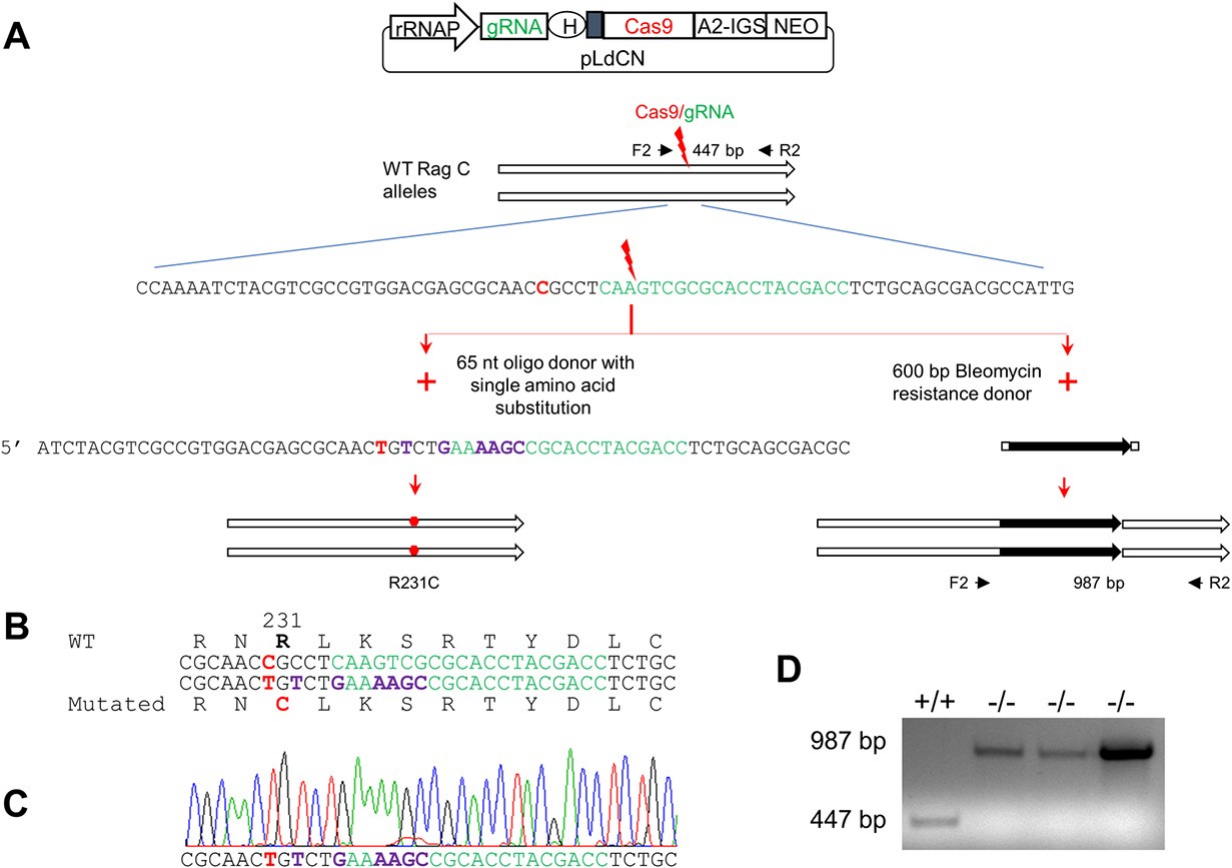
Generation of the RagC single amino acid substitution (R231C) mutant and RagC disruption mutant by CRISPR/Cas9. **A**. Strategy used to generate the RagC R231C mutant and *RagC* gene disrupted strains. A gRNA was designed to target a site (green) close to the R231C polymorphism (red) identified in the *RagC* gene of the Sri Lankan cutaneous *L*. *donovani* isolate. *L*. *donovani* 1S2D promastigotes were transfected with a CRISPR vector (pLdCNld366140) expressing this *RagC* specific gRNA, followed by transfection of the donor repair template which contained either: the targeted point mutation (C/T, red) and an additional six nucleotides resulting in silent mutations (purple) to protect the repaired genome from subsequent Cas9 cleavage, or a bleomycin selection marker (black). Genomic DNA from these *L*. *donovani* cells clones was subjected to PCR and sequencing analysis. **B.** Partial sequence of the oligo donor repair induced mutations resulting in a single amino acid substitution in RagC protein (R231C, shown in red) and inactivation of the gRNA targeting site (shown in green, interspaced with disrupting silent mutations in purple). **C.** Direct sequencing of a PCR product amplified from a *L*. *donovani* clone showing both alleles of the *RagC* gene have been edited to the sequence of the oligo donor (see A & B) repair template. **D.** PCR analysis of *RagC* double allele gene disrupted mutants. PCR analysis of three phleomycin resistant clones with primers F2 and R2 show the Bleomycin resistance gene has been inserted into the target site as expected resulting in a 987 bp band, and no 447 bp WT F2R2 band was detected in these *RagC* disruption mutants.

We initially focused on the RagC single amino acid mutation (R231C) since this mutation is naturally occurring in the atypical cutaneous strain of *L*. *donovani* in Sri Lanka [7,8]. Using CRISPR gene editing, an identical single amino acid mutation was introduced into both chromosomal copies of the *RagC* gene in the virulent *L*. *donovani* 1S2D strain as outlined in Fig 2A and 2B. To engineer the missense mutation, a gRNA with its targeting site close to the polymorphism site was designed. A single strand oligonucleotide donor DNA flanking the Cas9 cleavage site was synthesized with the desired C to T point mutation along with several synonymous mutations to protect edited genomes from further Cas9 cleavage (Fig 2A and 2B). RagC missense mutation containing clones were isolated after transfection of the donor DNA as previously described [20,21]. The heterozygous (231^R/C^) clones were first identified by using donor specific PCR. Since the Cas9 continued scanning the genome and generated the specific double-strand DNA break, the homozygous RagC R231C mutant was subsequently isolated from one of these heterozygous clones and confirmed by DNA sequencing (Fig 2C). In addition to generating the RagC R231C single amino acid mutant, the *RagC* gene was functionally disrupted by the insertion of a bleomycin resistance gene into the Cas9 cut site as also shown in Fig 2A and 2D.

### Effects of the RagC mutations on *L*. *donovani* promastigote proliferation and infection in BALB/c mice

**Fig 3:**
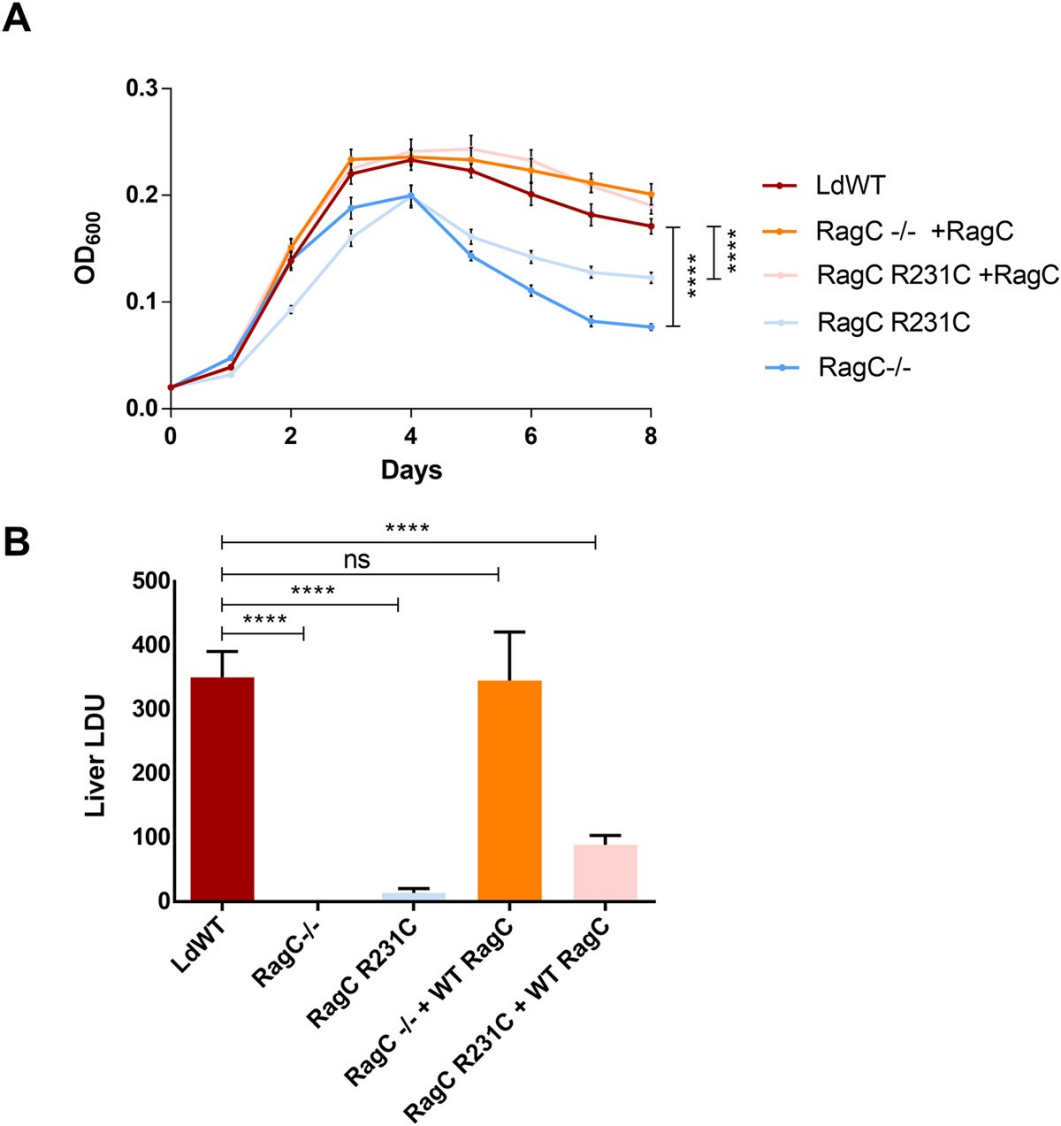
Biological effects of a Rag C single amino acid change (R231C) and disruption of the RagC gene. **A.** Proliferation of RagC WT (red), gene disrupted (-/-) (blue) and gene edited (R231C) strains (light blue), and RagC WT addback transfections in gene disrupted (orange) and gene edited (pink) strains. Data was measured in quadruplicate and statistical significance calculated using 2way ANOVA with multiple comparisons using RagC WT as the control group and marked for significance if consistent for every time point past day 2. This is the representative data of four repeat experiments. **B.** Effects of a single amino acid change (R231C) and disruption of *RagC* on *L*. *donovani* 1S2D infection in mice. BALB/c mice were infected by tail vein injection (1x10^8^ pro/mouse) with *L*. *donovani* WT, gene edited RagC R231C, gene disrupted *RagC* (RagC-/-), and their corresponding RagC WT addback strains. Mice were examined for liver parasite burden four weeks after infection. Data was measured using 4 mice per group and statistical significance calculated using 2way ANOVA with multiple comparisons using RagC WT as the control group.

As the TOR pathway plays a fundamental role in regulating cell proliferation in mammalian eukaryotes, we examined whether the RagC R231C point mutation and the *RagC* gene disruption mutation affected *L*. *donovani* 1S2D proliferation under culture conditions. As shown in the sand fly stage promastigote culture (27 ^o^C, pH 7.0), both the RagC R231C and *RagC* gene disrupted mutants were viable but could not reach the same cell density at day 4 as the wildtype *L*. *donovani* 1S2D (Fig 3A). Re-introducing a plasmid derived wildtype *RagC* gene to the mutant strains restored their proliferation to a level similar to that of the wildtype *L*. *donovani* 1S2D promastigotes. These results show the point mutation of the *RagC* gene was similar to disruption of the *RagC* gene and generated viable promastigotes that were unable to reach the same cell density as wildtype promastigotes. Interestingly, although both the RagC mutants showed reduced growth in culture, their ability to generate metacyclic like cells was not impaired (S1 Table).

We next determined whether the RagC R231C mutation or gene disruption mutant affected *L*. *donovani* parasite survival during visceral infection. The RagC R231C mutant and gene disruption mutant were injected into the tail vein of BALB/c mice. The liver parasite burdens usually peak at 4 weeks post infection in the BALB/c mouse model for visceral *Leishmania* infection and decrease subsequently due to development of immunity in the infected mice. The liver parasite burdens were therefore determined 4 weeks post tail vein infection. As shown in Fig 3B, both the RagC R231C mutant and the *RagC* gene disruption mutant had dramatically reduced parasite burden in the liver compared to wildtype *L*. *donovani* 1S2D. Addback of the wildtype RagC to the *RagC* gene disruption mutant fully restored parasite numbers in the liver. Addback of wildtype RagC to the RagC R231C mutant only partially restored the infection levels in the liver possibly because of competition with the chromosomal derived mutant R231C RagC. Taken together, these data demonstrate that similar to the *RagC* gene disruption mutant, the RagC R231C single amino acid mutant was attenuated for visceral infection in the mouse liver compared to wildtype *L*. *donovani* 1S2D.

### *Leishmania* wildtype RagC and mutant RagC R231C interact with RagA

**Fig 4:**
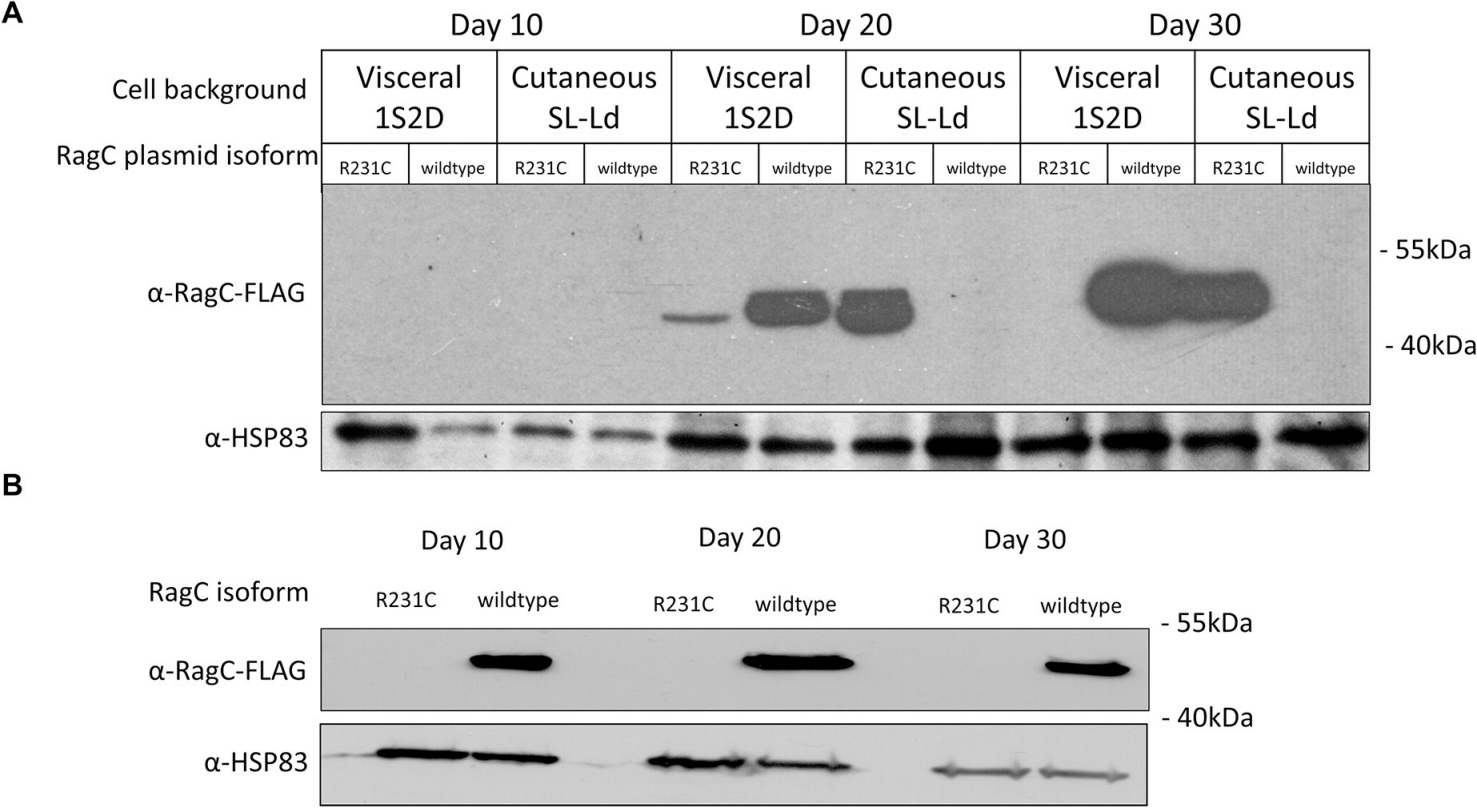
Incompatibility between the co-expression of wildtype and mutant RagC due to dominant negative effect. **A.** Immunoblotting of FLAG-tagged RagC wildtype and R231C mutant transfected in *L*. *donovani* 1S2D (Ld 1S2D) and cutaneous *L*. *donovani* from Sri Lanka (Cutaneous SL-Ld) strains followed over a period of 30 days. **B.** Immunoblotting of FLAG-tagged RagC wildtype and R231C transfected in *L*. *donovani* 1S2D cell following disruption of the endogenous *RagC* gene. The results are representative of three independent experiments.

Considering the bioinformatic analysis of the *Leishmania* TOR and upstream sensing pathways (S1 Fig), and the phenotypic impact of the RagC mutation (Fig 3), it was necessary to determine whether RagC interacts with RagA and whether the R231C single amino acid mutation alters this interaction. Plasmid vectors expressing epitope tagged versions of wildtype RagC, mutant RagC and RagA were transfected into *L*. *donovani* 1S2D and co-immunoprecipitation analysis carried out as detailed in Materials and Methods. Although it was possible to express the tagged wildtype RagC, it was difficult to obtain stable expression of the tagged RagC R231C mutant in *L*. *donovani* 1S2D. Further analysis revealed this was due to a selection against the transfected plasmid retaining the insert DNA encoding the mutant R231C RagC, although there was no selection against expressing wildtype RagC (S2 Fig). Plasmids expressing wildtype and mutant RagC were identical except for the R231C codon. Attempts were then made to express the RagC R231C mutant in the cutaneous *L*. *donovani* strain where this RagC mutant was first identified [8]. As shown in Fig 4A, at 20- and 30-days post-transfection, the cutaneous *L*. *donovani* strain (cutaneous SL-Ld) could support the expression of the mutant RagC R231C but selected against expression of the wildtype RagC and conversely, wildtype *L*. *donovani* 1S2D could support the expression of the wildtype RagC but not the mutant RagC R231C. We further attempted to express the RagC R231C mutant in *RagC* gene disrupted 1S2D, however as shown in Fig 4B, *L*. *donovani* 1S2D devoid of a genomic copies of wildtype *RagC* selected against expressing the plasmid derived RagC R231C mutant. This is consistent with the above observation that expression of the RagC R231C mutant from the edited chromosome showed reduced cell density compared to wildtype *L*. *donovani* 1S2D (Fig 3A). It is possible that mutant RagC R231C produces a dominant negative effect resulting in the strong selection against plasmids expressing mutant RagC R231C in *L*. *donovani* 1S2D.

**Fig 5:**
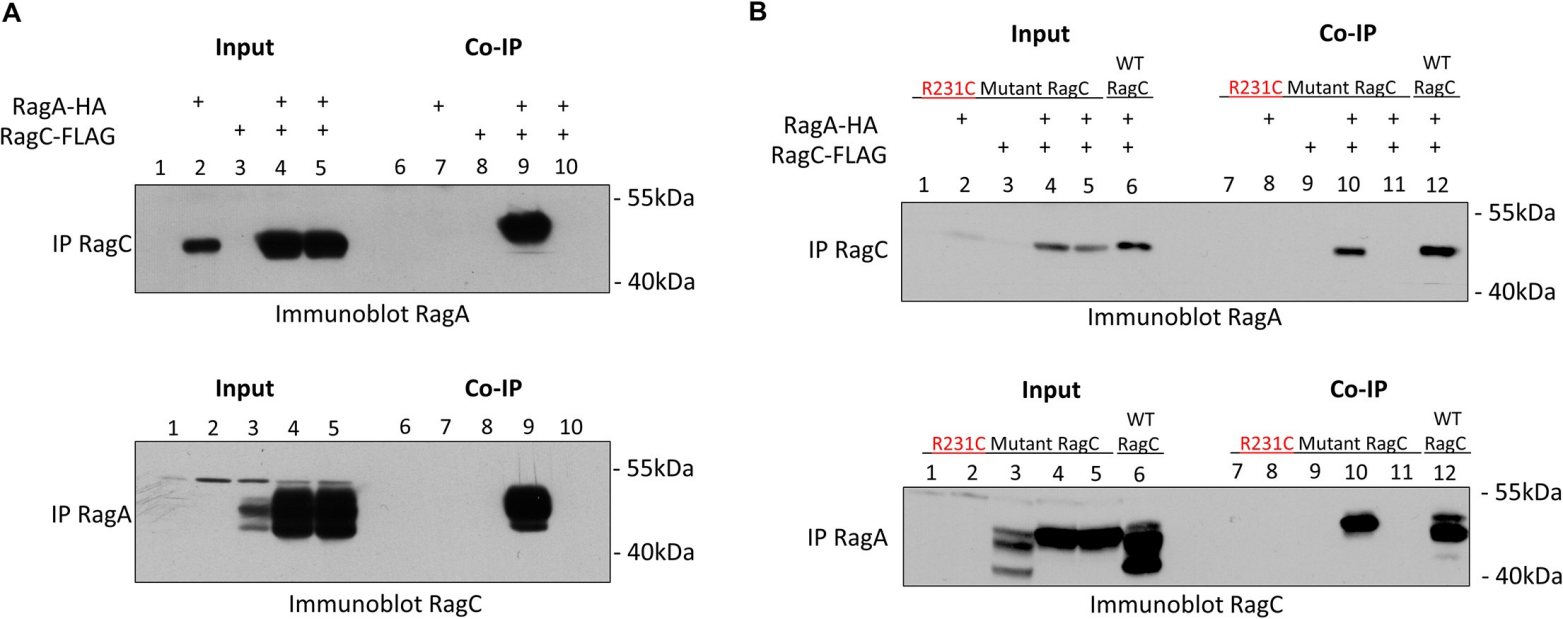
*Leishmania* RagA and RagC proteins form a heterodimer complex. **A**. Heterodimer formation between wildtype RagC and RagA. Top Panel: RagC was immunoprecipitated with FLAG antibodies and immunoblotting with HA antibodies shows the presence of HA-tagged RagA (Lanes 9) specifically in RagC co-transfected cells. Western blot analysis of input lanes 1–5 are also shown. Bottom Panel: Co-immunoprecipitation showing RagC is captured following immunoprecipitation of RagA. HA-tagged RagA was immunoprecipitated followed by immunoblotting with FLAG antibodies shows the presence of RagC (Lane 9) specifically in Rag A co-transfected cells. Western blot analysis of input lanes 1–5 are also shown. **B.** Heterodimer formation between mutant R231C RagC and RagA. Top panel: RagC R231C was immunoprecipitated using FLAG antibodies followed by immunoblotting with HA antibodies shows the presence of HA-tagged RagA in RagC R231C co-transfected cells (Lane 10 and positive control Lane 12). Western blot analysis of input lanes 1–6 are shown. 5B Bottom Panel: HA-tagged RagA was immunoprecipitated using HA antibodies followed by immunoblotting with FLAG antibodies shows the presence of FLAG-tagged RagC (Lane 10 and positive control Lane 12). Western blot analysis of input lanes 1–6 are shown. The results are representative of three independent experiments.

Considering the above observations, the interaction between wildtype RagC and RagA was initially investigated in transfected *L*. *donovani* 1S2D promastigotes. As shown in Fig 5A, lane 9, following immunoprecipitation of FLAG-tagged RagC, it was possible to detect RagA by immunoblotting with HA antibodies. Control lanes showed that RagA was not pulled down in the absence of RagC (Fig 5A, Top Panel, lane 7) or if anti-FLAG antibodies were not present (Fig 5A, Top Panel lane 10). In the reverse, following immunoprecipitation of HA-tagged RagA, it was possible to detect RagC following immunoblotting with FLAG antibodies (Fig 5A, Bottom Panel, lane 9). Control lanes showed that RagC was not detectable in the absence of RagA (Fig 5A, Bottom Panel, lane 8) or if anti-HA antibodies were not present (Fig 5A, lane 10). Further, epifluorescence microscopy using GFP tagged RagA and mCherry tagged RagC revealed that both proteins localized to overlapping nuclear-adjacent foci on the kinetoplast pole of the promastigote as shown in S3A Fig, indicating the proteins co-localize *in vivo*. These results show that wildtype RagC and RagA can form a heterodimer or are present in the same complex consistent with the *in silico* bioinformatic analysis shown in **S1 Fig**.

We next determined whether the R231C mutation in RagC impaired the interaction with RagA when both proteins were co-expressed in the cutaneous strain of *L*. *donovani* that was permissive for expression of transfected RagC mutant R231C. As shown in Fig 5B, following immunoprecipitation of the RagC R231C mutant with FLAG antibodies, it was possible to detect RagA following immunoblotting with HA antibodies (Fig 5B, Top Panel, Lane 10) at comparable levels to immunoprecipitation of wildtype RagC expressed in *L*. *donovani* 1S2D (Fig 5B, Top Panel, Lane 12). Control lanes showed that RagA was not pulled down in the absence of RagC R231C mutant (Fig 5B, Top Panel, lane 8) or in the absence of FLAG antibodies (Fig 5B, Top Panel, lane 11). In the reverse, following immunoprecipitation of HA-tagged RagA, it was possible to detect the RagC R231C mutant following immunoblotting with FLAG antibodies at comparable levels to wildtype RagC expressed in *L*. *donovani* 1S2D (Fig 5B, Bottom Panel, lanes 10 and 12). Control lanes showed that the RagC R231C mutant was not detectable in the absence of RagA (Fig 5B, Bottom Panel, lane 9) or in the absence of HA antibodies (Fig 5B, Bottom Panel, lane 11). Further, epifluorescence microscopy showed GFP tagged RagA and mCherry tagged RagC R231C mutant also localized to overlapping nuclear-adjacent foci (**S3**B Fig). These results provide evidence that the R231C mutation in RagC did not impair its ability to interact with RagA or alter the cell location of the RagA-RagC heterodimer under these experimental conditions.

As the RagC R231C mutant was able to interact with RagA, we used *in silico* analysis to further investigate how the mutation could affect the structure of RagC. The amino acid sequences for mutant RagC were subjected to homology modelling using a human template as detailed in Materials and Methods. As shown in S4A Fig, the R231C mutation (red) is located away from the RagA (yellow) and RagC (blue) proposed interaction site based on human RagA and RagC crystal structures [22], indicating this mutation is unlikely to significantly affect the interaction between these proteins, consistent with the results shown in Fig 5. In addition, the point mutation is not in the GTPase domain suggesting this does not affect enzyme activity. We did however identify an extra loop of amino acids forming a new motif on the surface of RagC in *Leishmania* not present in human and yeast proteins as shown in the alignment in S4B Fig and in the ribbon representation the new motif is highlighted in green (S4C Fig).

Bioinformatic analysis of mTORC1 complex proteins revealed not only the conservation of the *Raptor* gene but also a single amino acid mutation (A969E) between the cutaneous and visceral strains of *L*. *donovani* (S1 Fig) [7]. Since Raptor interacts with the RagC/RagA heterodimer in mammalian cells and plays a key role in mTORC1, we investigated whether introducing this *Raptor* gene mutation in wildtype *L*. *donovani* 1S2D (S5A Fig) could make these cells permissive for the expression of the R231C RagC. Although this single amino acid mutation was successfully edited in the chromosome using CRISPR, this mutation alone could not reverse the selection against expression of the R231C RagC in the *L*. *donovani* 1S2D strain (S5B Fig).

### The *RagA* gene is essential for *Leishmania* promastigotes

**Fig 6:**
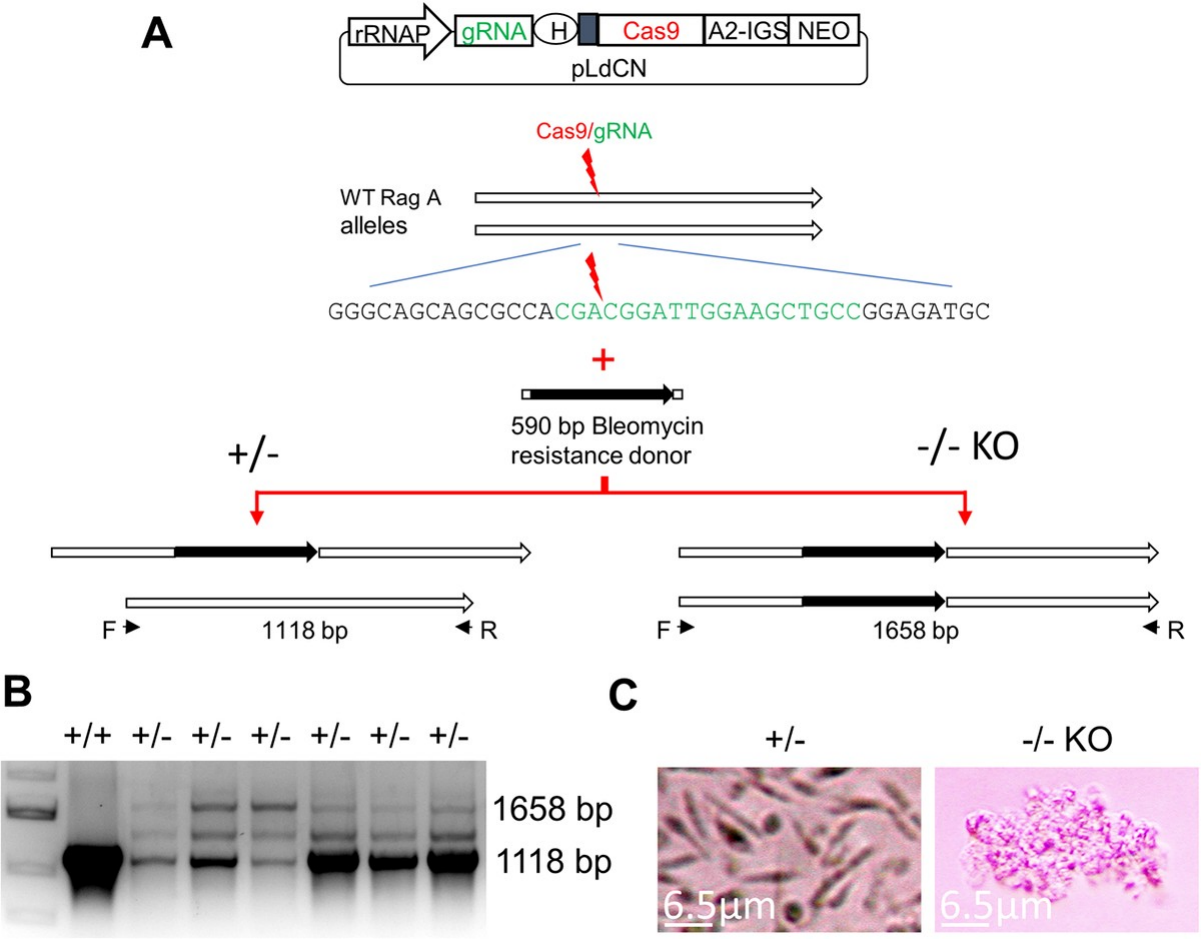
*RagA* is essential for Leishmania. **A**. Strategy used to generate the single allele or double allele *L*. *donovani RagA* disruption mutants. A gRNA was designed to target the first half of the RagA coding sequence. *L*. *donovani* 1S2D cells were transfected with a CRISPR vector (pLdCNld131620) expressing this *RagA* specific gRNA, followed by transfection of the bleomycin selection marker donor with 25 nt flanking sequences to integrate into the Cas9 cut site. **B.** PCR analysis of the surviving phleomycin resistant clones showing the bleomycin resistance gene was inserted into the target site of one *RagA* allele as expected, but the 1118 bp WT *RagA* band was still detected in all these phleomycin resistant clones. *Note*: *the middle band is the annealing product during PCR between the 1118 bp WT RagA band and the 1658 bp disruption band*. **C**. Microscope images showing the disruption of both *RagA* alleles is lethal for *L*. *donovani*. The *RagA*+/- partial mutant cells expressing the *RagA* targeting gRNA were cloned in a 96 well plate and cell growth was monitored by microscopy. The image for *RagA* +/- cells was taken one week after cloning; The image for *RagA*-/- cells was taken four weeks after cloning.

In yeast and mammalian cells, it is possible to generate single and double *RagA* and *RagC* null mutants showing both these proteins are non-essential in these eukaryotic cells [9,16,23,24]. We therefore investigated whether it was possible to disrupt the *RagA* gene in *L*. *donovani* 1S2D and to compare the resulting phenotype to the *RagC* disruption mutant. The experimental approach to disrupt the *RagA* gene in *L*. *donovani* 1S2D is shown in Fig 6A. While it was possible to generate promastigotes with a single *RagA* allele disrupted, one copy of the wild type *RagA* allele persisted in the surviving *Leishmania* promastigotes (Fig 6B, lower band). Following single cell cloning of surviving promastigotes to isolate double allele knockout cells, the *RagA* double null mutant clones continued replicating to approximately one hundred cells before dying while single allele *RagA* disrupted parasites continued to proliferate (Fig 6C). It was therefore not possible to establish a culture with a double *RagA* gene disruption and thus was not possible to isolate DNA for PCR analysis. Together, this demonstrates that *RagA* is essential for *Leishmania* promastigotes survival.

## Discussion

A major objective of this study was to investigate the role of a naturally occurring single amino acid mutation, R231C in RagC, in contributing to the attenuation of *L*. *donovani* in Sri Lanka for causing visceral disease. CRISPR gene editing was used to change this single amino acid in the RagC protein in the background of wildtype virulent *L*. *donovani* 1S2D. The observations presented in experimentally infected mice provide evidence that this naturally occurring RagC mutation in the cutaneous *L*. *donovani* strain from Sri Lanka [7,8] contributed to its attenuation for infection in visceral organs. It is however unknown whether this mutation also contributes to the ability of this atypical strain to cause cutaneous infections as is widespread for *L*. *donovani* in Sri Lanka [6]. It is likely that in addition the *RagC* mutation, some of the additional 91 mutations identified in the cutaneous strain [7,8] are necessary for survival in cutaneous sites and stable expression of the RagC R231C protein isoform.

Using CRISPR gene editing, it was possible to investigate the biological outcome of the RagC R231C mutation against a wildtype virulent *L*. *donovani* genetic background in the absence of the other 91 gene mutations in the atypical *L*. *donovani* cutaneous strain [7]. It is noteworthy that the independent *RagC* gene disruption mutation had a similar phenotype in promastigotes and in infected mice as the RagC R231C mutation and in both mutants this was reversed in the add back clones further supporting the argument for the importance of the *RagC* gene for *L*. *donovani* visceral infection. It is remarkable that a single homozygous SNP resulting in RagC R231C can have such a dramatic effect on parasite numbers in the liver in experimentally infected mice. It is unknown what effect these mutations have on differentiation in the sand fly such as for example the development of metacyclics. It is however unlikely that a *Leishmania* mutant with impaired metacyclic development would be viable in nature. This is consistent with the observation that, if anything, there was an increase in the number of metacyclics in both the R231C RagC point mutant and the *RagC* gene disruption mutant promastigote stationary phase cultures. Future studies are needed to investigate the increased metacyclic formation in the RagC mutants.

A secondary objective of this study was to investigate components of the *Leishmania* TOR signaling and upstream sensing pathways that includes RagC. *In silico* bioinformatic analysis suggested that similar to mammalian cells, *L*. *donovani* retains several key components of the TOR sensing pathway including RagC, RagA and several components of the TORC1 complex including Raptor (S1 Fig). Immunoprecipitation analysis demonstrated that RagC can form heterodimers with or is in the same complex as RagA which experimentally supports the *in silico* analysis. Attempts to identify the cellular defect resulting from the RagC mutations were however unsuccessful since the mutant RagC retained the ability to interact with RagA (Fig 5) and introduction of the *Raptor* A969E gene mutation by gene editing did not make *L*. *donovani* 1S2D permissive for expression of transfected mutant RagC (S5 Fig). Further, The RagC mutation did not appear to affect the localization of the heterodimer complex as both wildtype and mutant proteins were shown to co-localize in perinuclear foci (**S3 Fig**). This localization pattern is consistent with the localization of the *Leishmania* homologue to Rab7 [25,26], which in mammalian cells is present in the same lysosomal compartment as the RagA/RagC (or RagB/RagD) complexes [17]. One possibility consistent with these observations is that the mutant RagC/RagA interaction abnormally controls the activity of downstream components of the TOR pathway (perhaps mTORC1 itself) resulting in attenuation of *L*. *donovani* for survival in visceral organs.

Several sensor proteins upstream of and other regulators of the RagA/RagC complex present in mammalian cells appear to be missing from *Leishmania* including RagB or RagD proteins (Figs 1 and S1, Table 1), suggesting the RagA/RagC complex plays a more central role in *Leishmania* signaling than in mammalian cells. This is consistent with the observation that it was not possible to disrupt both alleles of the *RagA* gene in *L*. *donovani* (Fig 6) although this is possible in mammalian and yeast cells [9,16,23,24]. Although the R231C RagC retained the ability to interact with RagA, wildtype *L*. *donovani* 1S2D could not tolerate the expression of the mutant R231C RagC (Fig 4) likely due to the mutant having a dominant effect. Presumably, other mutations or alterations in the cutaneous *L*. *donovani* strain enable the expression of the R231C RagC through epistasis. Indeed, there is also a *Raptor* A969E gene mutation in the atypical cutaneous *L*. *donovani* strain although introduction of this mutation in *L*. *donovani* 1S2D did not enable expression of the R231C RagC (S5 Fig). One or more of the 91 altered genes previously identified in the cutaneous *L*. *donovani* strain [7] may be necessary to enable expression of the R231C RagC protein. Future studies could include performing pull down immunoprecipitation and mass spectrometry to compare mutant R231C RagC and wildtype RagC protein interactions. It will also be interesting to investigate whether the R231C RagC mutant or the Raptor A969E mutant affect the response to different nutrients or the recruitment of mTORC1 proteins to the lysosomes and activation of downstream signaling pathways. Interpretation of these experiments could however be compromised because of the necessity to express wildtype and mutant epitope-tagged RagC proteins in different *L*. *donovani* strain backgrounds as demonstrated here.

In mammals, leucine, arginine, and glutamine have been shown to trigger mTORC1 activity. The CASTOR1/2 protein complex and solute carrier family38 member 9 (SLC38A9) (a lysosomal amino acid transporter) were identified as intracellular arginine sensors [14,18,27].Our bioinformatic analysis has shown that *Leishmania* has the homologs for SLC38A9 and components for the GATOR2 complex, normally downstream of CASTOR1/2 but we did not identify any proteins with significant CASTOR homology in *Leishmania*. It has been recently reported that in order to survive arginine deprivation in macrophages, *Leishmania* up regulates expression of its arginine transporter (AAP3) through a MAPK2-mediated arginine deprivation response (ADR) pathway [28]. As the identity of the arginine sensor that triggers the ADR pathway is unknown, it will be interesting to investigate whether the ADR pathway shares the same potential arginine sensors (SLC38A9 and a possibly unidentified protein upstream of GATOR2) with mTORC1 signaling pathway and whether the ADR pathway is downstream of or parallel to the mTORC1 pathway. It will also be of interest to determine whether the potential arginine sensors can directly interact with the RagA-RagC heterodimer and whether the RagC R231C mutation interferes with interactions between these upstream amino acid sensors and the RagA-RagC heterodimer.

It was not possible to detect wildtype RagC protein expression in the transfected cutaneous *L*. *donovani* promastigotes. This is not consistent with our previous observation where transfection of the wildtype *RagC* gene in the cutaneous *L*. *donovani* isolate increased parasite numbers in the spleen of BALB/c mice following tail vein injection, albeit at a much lower level than wildtype *L*. *donovani* [8]. Since RT-PCR did show the presence of wildtype *RagC* transcripts in the transfected cutaneous *L*. *donovani* isolate [8], it is possible that although the transfected cutaneous strain promastigotes in culture had selected against expression of wildtype RagC, a small minority of promastigotes retained expression and these promastigotes then gained a survival advantage once present in the mouse visceral organs.

Although it was possible to generate a disruption mutation in the *RagC* gene, disruption of the *RagA* gene in *L*. *donovani* 1S2D resulted in promastigote death. *RagA* can therefore be considered an essential gene and plays an important role in *L*. *donovani* viability while RagC potentially plays a more regulatory role. Indeed, it has been observed that in human cells, the RagA protein is responsible for the initial interaction with the TORC1 complex and that RagC stabilizes this interaction [29] and that RAPTOR is preferentially sensitive to the GTP/GDP state of RagA and not RagC [9,16]. This could help explain our observations that RagC but not RagA was dispensable in *L*. *donovani* promastigotes.

A recent genome sequence analysis of 8 additional *Leishmania* parasites from Sri Lanka revealed that all were indeed *L*. *donovani* but were genetically distinct from each other [30] and different from the cutaneous *L*. *donovani* strain initially sequenced [7,8] and under investigation in this study. This demonstrates that there is a high level of diversity in the atypical *L*. *donovani* strains currently circulating in Sri Lanka. Analysis of the deposited GenBank sequences for these additional genomes [30] revealed that none had the RagC R231C or Raptor A969E mutations described here nor did they have any of the other nonsynonymous SNPs previously identified in the original cutaneous *L*. *donovani* strain [7,8]. This is interesting because it reveals that there are multiple genotypes of *L*. *donovani* in Sri Lanka yet virtually no cases of visceral leishmaniasis [6]. Although the RagC R231C mutation may contribute to the attenuated visceral phenotype in the *L*. *donovani* strain under investigation here [7,8], it appears that other atypical strains in Sri Lanka became attenuated through alternative mechanisms [30]. The observations from this study nevertheless provide evidence that perturbation of the conserved sensing and TOR pathways in *L*. *donovani* can influence human pathogenesis.

## Materials and methods

### Ethics statement

The animal protocol was approved by the McGill University Faculty of Medicine Animal Care Committee, Protocol Number: 2012–7112.

### BLAST searches

Comparison of the human and *L*. *donovani* TOR pathways was generated using human protein sequences obtained from UniProt in FASTA format [31]. The protein sequences were then searched for using the BLASTP [32] application accessed through TriTrypDB [33] using the *Leishmania donovani* strain LdCL [7] database. The sequences were also compared to the available protein sequences for all *L*. *major*, *L*. *infantum*, *L*. *tropica*, *L*. *mexicana* and *L*. *braziliensis* strains available on TriTrypDB. For all searches, an E-value cut-off of 1.0 was used to filter out false positive low scoring alignments.

### Leishmania strain and culture media

*L*. *donovani* 1S2D strain used in this study were routinely cultured at 27°C in M199 medium (pH 7.4) supplemented with 10% heat-inactivated fetal bovine serum, 40 mM HEPES (pH 7.4), 0.1 mM adenine, 5 mg l^−1^hemin, 1 mg l^−1^ biotin, 1 mg l^−1^ biopterin, 50 U ml^−1^ penicillin and 50 μg ml^−1^ streptomycin. Cultures were passaged to fresh medium at a 40-fold dilution once a week. The growth curves of *L*. *donovani* RagC (R231C) mutant and null mutant in promastigote culture medium were obtained by inoculating the parasites in 1 x 10^***6***^ / ml into the 96 well plate **(**180 μl/well) in quadruplicate, the OD values were measured once a day for eight days

### Negative selection of metacyclic promastigotes with peanut agglutinin (PNA)

The metacyclic promastigotes were isolated with PNA as described [34]. Briefly, the densities of healthy *L*. *donovani* stationary phase promastigotes was first determined by light microscopy. The promastigotes were then harvested at 2,000 g for 5 min and resuspended at a cell density of 2 x 10^8^ cells/ml in complete medium containing 50 μg/ml PNA. Promastigotes were allowed to agglutinate at room temperature for 30 min. The supernatants were then carefully transferred to new tubes. The PNA negative promastigotes from these supernatants were counted using microscopy.

### CRISPR plasmid construction

The *L*. *donovani* RagC R231C mutant and the gene disrupted *RagC* and *RagA* using CRISPR/Cas9 gene editing technique were generated as described [20,35]. The addback RagC expression plasmid was generated by cloning the full RagC coding sequence into the *Hind* III and *BamH* I sites of the *Leishmania* expression plasmid pLphyg2 [36]. The RagC expression plasmid with a FLAG tag was generated by cloning the coding sequence with C-terminal tag into the *Hind* III and *BamH* I sites of *Leishmania* expression plasmid pLphyg2 [36] The RagA-HA tag expression plasmid was generated by first cloning the full RagA coding sequence into pLpneo2, followed by the insertion of an HA tag in frame.

The oligos and primers used in this study are listed below.

The following primers and oligos were used for generating the *L*. *donovani* RagC R231C mutant, the disruption mutant and for constructing the addback RagC expression plasmid with FLAG tag at its C terminus.

Oligos used to insert the *RagC* targeting gRNA into the pLdCN CRISPR vector:

Ld366140+ 5’ TTGTGGTCGTAGGTGCGCGACTTG

Ld366140- 5’ AAACCAAGTCGCGCACCTACGACC

R231C mutation containing repair donor oligonucleotide

Ld366140donor 5’ ATCTACGTCGCCGTGGACGAGCGCAACTGTCTGAAAAGCCGCACCTACGACCTCTGCAGCGACGC

Primers used to generate the Bleomycin resistance donor:

Ld366140BleF1 5’ CGTCGCCGTGGACGAGCGCAACCGCCTCAAGATCTTCATCGGATCGGGTA;

Ld366140BleR1 5’ GCGTCGCTGCAGAGGTCGTAGGTGCGCGACGTCGGTCAGTCCTGCTCCT.

Primers for PCR to verify the CRISPR generated point mutations and the disrupted mutants: Ld366140F2(F2 in Fig 2) 5’ CTGCTGCAGATGCTCAACTC

Ld366140R2(R2 in Fig 2) 5’ CACGTTACGGTCAATCAACG

Ld366140dF 5’ AGCGCAACTGTCTGAAAAGC

Primers used to construct the RagC (WT or R231C) expression plasmids with FLAG tag at its C terminus:

Ld366140F 5’ CCCAAGCTTCACTACTTGCTCGCCCTTT

Ld366140F1 5’ CCCAAGCTTCCGAGCATGTCCAACAACATGCTCGCACT

Ld366140R 5’ CCGAGATCTTGCGTGCCTCTCTCTCTCT

Ld366140R1(3xFLAG)5’ GATCCTTGTAGTCTCCGTCGTGGTCCTTATAGTCTGGATCCCGGGATGCACTCGTGTTGAAGATG

Bgl2FLAG 5’ CCGAGATCTACTTATCGTCATCGTCTTTGTAATCAATATCATGATCCTTGTAGTCTCCGTCGTGG

The following primers and oligos were used for generating the *L*. *donovani* Raptor (LdCL_250011400) A969E mutant:

Oligos used to insert the gRNA guide coding sequences into pLdCN vector:

LdRptM+ 5’ TTGTCGACGGTGTGCTGGTGAACA

LdRptM- 5’ AAACTGTTCACCAGCACACCGTCG

LdRptMdonor 5’ TCTACGCAAACTGCGACGGTGTGCTCGTCAATAAAGAACTGCGCTACCTCTCAAACGGC GCTC.

Primers for PCR to verify the CRISPR generated point mutations:

LdRptMdonF 5’ GGTGTGCTCGTCAATAAAGAA

LdRptML 5’ TTGACAGCCGATGATCCACT

LdRptMR 5’ TGACTGCTCTGAAAGGTCGT

The following primers and oligos were used for generating the *L*. *donovani RagA* disruption mutant and for constructing the RagA expression plasmid with an HA tag at its C terminus.

Oligos used to insert the gRNA guide coding sequences into pLdCN vector

Ld131620+ 5’TTGTGGCAGCTTCCAATCCGTCG

Ld131620- 5’ AAACCGACGGATTGGAAGCTGCC

Primers used to generate the Bleomycin resistance donor:

Ld131620BleF1 5’ GTTGGTGGCGAGGGCAGCAGCGCCACGATCTTCATCGGATCGGGTA

Ld131620BleR1 5’ CTCCAGCATCTCCGGCAGCTTCCAATCCGTCGGTCAGTCCTGCTCCT

Primers used to construct the RagA expression plasmids with an HA tag at its C terminus: Ld131620F (F in Fig 6) 5’ CCCAAGCTTCCACGCATGATTCTTCCGCGCTA

Ld131620R (R in Fig 6) 5’ CCGGGATCCAGAACTTCGCGCATTGCACAC

HAtag+ 5’ GATCTGTACCCATACGATGTTCCAGATTACGCTT

HAtag- 5’ GATCAAGCGTAATCTGGAACATCGTATGGGTACA

Ld131620F2 5’GCATGAACAACACGACCAGT

### Leishmania transfection and single cell cloning assay

*Leishmania* transfections were performed as previously described [20]. Briefly, 10 μg pLdCN CRISPR plasmid DNA encoding the specific gRNA was electroporated into 1 x 10^8^ early stationary phase *L*. *donovani* promastigotes. The transfected cells were then selected with G418 (50–100 μg /ml). To generate the RagC (R231C) mutant, once the pLdCN plasmid transfected *L*. *donovani* culture was established, the cells were subjected to three rounds of sequential oligonucleotide donor transfection, 10 μl (100 μM) single strand oligonucleotide donor was used per transfection, once every three days. After the third oligonucleotide donor transfection, the *Leishmania* promastigotes were counted and inoculated into 96 well plates in one promastigote per 100 μl medium per well. The growth of *Leishmania* cells in 96 well plates were monitored under microscope. After culture for three weeks in 96 well plates, parasites from the relatively slow growing clones were expanded in 24 well plates. The genomic DNA extracted from these slow growth clones were subjected to PCR and sequencing analysis. To generate the *RagC* or *RagA* disruption mutant, the *L*. *donovani* cells containing the corresponding pLdCN plasmid were subjected to transfection of the Bleomycin resistance gene donor PCR product and selected with 100 μg/ml phleomycin. The phleomycin resistant cells were then cloned into 96 well plates and monitored for growth under a microscope. After culture for three weeks in 96 well plates, the surviving parasites were expanded in 24 well plates and subjected to PCR analysis.

### Infection of mice with L. donovani RagC mutants

To determine how the RagC R231C amino acid change and disruption of the *RagC* gene would affect *L*. *donovani* virulence in visceral infection in BALB/c mice, mice were infected with both *L*. *donovani* RagC mutants and the wildtype *L donovani* 1S2D by tail vein injection with the same number of stationary phase promastigotes (1x10^8^ pro/mouse). Mice were examined for the liver parasite burden (*L*. *donovani* units, LDU) four weeks after infection as previously described [8].

### Co-immunoprecipitation

For each lane, 1x10^8^ cells were isolated and lysed on ice for 30 mins in 100 μL of lysis buffer (50 mM Tris pH 7.4, 150 mM NaCl, 0.25% NP-40) supplemented with one tablet of cOmplete ULTRA protease inhibitor cocktail (Roche). Note: As the cutaneous *Leishmania donovani* strain expressed plasmid encoded proteins to a lower level than the wildtype strain 1S2D, 5X more lysate was used as input in cutaneous *Leishmania* co-IP experiments. Lysate was clarified by centrifugation at 21,000x g for 20 mins. 10 μL of clarified lysate was mixed with SDS Sample Buffer and used as ‘Input’ lanes. 1 μL of FLAG or HA antibody was added to each tube of clarified lysate and the reaction volume adjusted to 500 μL with Lysis Buffer. Lysate:Antibody mixture were incubated for 2h at RT with gentle agitation on a tube revolver. For each tube, 25 μL of Protein A/G Magnetic beads (Pierce) were washed in Lysis Buffer for 5 mins and added to the mixture. Tubes were incubated for an additional hour at RT on a tube revolver. Beads were isolated from the tubes using a DynaMag2 magnetic stand (Life Technologies). The beads were then washed three times with 1 mL of Lysis Buffer and resuspended fully each time. Captured and bait proteins were eluted off the beads by incubating in 2X SDS Sample Buffer for 10 mins followed by SDS-PAGE.

### Immunoblotting

RagC-FLAG: Input and Co-IP samples were prepared as described above subjected to SDS-PAGE. Primary mouse IgG against FLAG tag (M2 clones, Sigma) was used at 1:10,000 diluted in PBS-T with 5% skim milk powder. Secondary HRP labelled goat IgG against mouse IgG (GE Healthcare) was used at 1:10,000 diluted in PBS-T with 5% skim milk powder. ECL reagent (ZmTech Scientific) was incubated for 2 minutes and the membranes were exposed to X-Ray film (Denville). Resulting film was imaged on a FluorChem FC8800 gel imager (Alpha Innotech).

RagA-HA: Immunoblotting was carried out as described above with rabbit HA antibodies (EMD Millipore) and HRP conjugated donkey anti-rabbit IgG antibodies (ThermoFisher Scientific).

### Epifluorescence microscopy

The RagA-GFP expression plasmid was created by cloning the full *L*. *donovani* RagA coding sequence into the *Hind* III and *BamH* I restriction sites of the pLGFPN plasmid [37]. The RagC-mCherry expression plasmid was created by using NEBuilder HiFi (New England Biolabs) to insert the mCherry coding sequence into the *Hind* III restriction site of the plphyg2-RagC plasmids. WT and Sri Lanka cutaneous *L*. *donovani* parasites were transfected with both plasmids and selected with a combination of hygromycin and neomycin. 10 μL of cell suspension was diluted into 100 μL of PBS and cytospun on poly-lysine coated cover slips. The cells were fixed with 4% paraformaldehyde for 10 minutes and blocked with 5% BSA in PBS for 30 mins. The coverslips were mounted on glass slides with a drop of Prolong Antifade Glass (Invitrogen) and 2 μL of 10 μM DRAQ5 (ThermoFisher Scientic) and left to cure overnight at RT in the dark. Slides were imaged the following day on an ImageXpress Micro Confocal high content screener (Molecular Devices) in widefield acquisition with MetaXpress software.

### Homology modelling

The RagA and RagC protein coding sequences were used to generate the homology models using the SWISS-MODEL protein homology server [38]. The best scoring human template was selected (ID 6CES [22]) for modeling. The resulting *Leishmania* RagA and RagC proteins were aligned over the human proteins in complex for orientation. The modeled proteins were then further relaxed using the Relax application of the Rosetta software [39–42]. Protein models were visualized and color coded using UCSF Chimera [43].

## Supporting information

S1 Table A table showing the percentages of metacyclic like promastigotes present in the stationary phase cultures of wild type L. donovani, R231C RagC mutant and the RagC null mutant. Comparison of the proportion of metacyclic cells present in stationary phase cultures.
(PDF)

S1 Fig Hypothetical model of the amino acid sensing Rag pathway in *Leishmania*. Components of the Rag pathway conserved in *Leishmania* based on sequence homology determined through bioinformatic analysis of the *Leishmania* genome. Note, there are several human components that were not identified in *Leishmania* including RagB and RagD. GATOR1 proteins were identified but with low homology or only partial member homology and are shown in grey. Also indicated are the RagC R231C, and Raptor A969E mutants in red.
(TIF)

S2 Fig PCR determination of insert size with the wildtype and mutant RagC protein plasmids. WT promastigotes were transfected with plasmids encoding either the WT or mutant copy of RagC. At 4 weeks post transfection the plasmids were recovered using a plasmid DNA mini-preparation kit. Primers (arrows) were designed to the C terminal end of the hygromycin resistance cassette (Hyg) and downstream of the protein insertion site (MCS) in order to target episomal copies of *RagC* only. The first lane products originate from an empty plasmid (Band type 2). The second lane shows an increase in band size due to the insertion and retention of the RagC protein on the plasmid (Band type 1). The third lane shows the excision of not only the RagC protein but the flanking UTR as indicated by the large decrease in size of amplicon (Band type 3).
(TIF)

S3 Fig Co-localization of RagA with RagC in *L*. *donovani*. **A.** Epifluorescence microscopy of RagA and RagC. GFP-tagged RagA and mCherry-tagged RagC form overlapping foci near the kinetoplast and nucleus in transfected promastigotes. **B.** Epifluorescence microscopy of RagA and RagC R231C. GFP-tagged RagA and mCherry-tagged RagC R231C mutant form overlapping foci near the kinetoplast and nucleus in transfected promastigotes.
(TIF)

S4 Fig Location of the RagC R231C mutation. **A.** Homology modelling of *Leishmania donovani* RagA (yellow) in complex with RagC R231C (blue) shown in ribbon representation. The 231^st^ amino acid position on RagC corresponding to the identified mutation is highlighted in red full atom representation and indicated by arrows. The complex is oriented with the Roadblock interaction domains shown on top and the GTPase domains at the bottom. **B.** Multiple sequence alignment between *Leishmania*, human and yeast RagC homologue sequences with *L*. *donovani* RagC R231 highlighted. C-terminal portion of the alignment shown with a 37 amino acid long sequence only seen in *Leishmania* highlighted **C.** Homology model of the RagA/RagC complex with the *Leishmania* specific sequence from B. shown in green.
(TIF)

S5 Fig Generation of 1S2D *Raptor* mutant parasites does not enable expression of RagC R231C. **A. S**trategy used to insert the Raptor A969E mutation into the *Raptor* gene of *L*. *donovani*. A gRNA was designed to target the *Raptor* gene at the site of the desired C to A base change and cloned into the CRISPR vector (pLdCNld251140) expressing this gRNA and Cas9 nuclease. This plasmid was transfected into 1S2D *L*. *donovani* cells followed by the transfection of a 63 nt donor oligonucleotide with 25 nt flanking sequences to introduce the desired C to A conversion (red) and 5 silent mutations to prevent further cleavage by Cas9 (purple). The cells were then cloned into 96-well plates and screened by PCR and Sanger sequencing. **B.** Immunoblot following the expression of RagC isoforms in the *Raptor* mutant parasites. The isolated parasites were transfected with plasmids expressing either the WT or R231C isoforms of RagC. At 10 days post-transfection, both isoforms are expressed at comparable low levels. At 20 days, the expression of the WT isoform is stabilized, the R231C isoform appears at slightly reduced levels compared to the 10-day time point. At 30 days post transfection, only the WT isoform is stably expressed.
(PNG)
