# Supplementary material for: Evidence that a naturally occurring single nucleotide polymorphism in the RagC gene of Leishmania donovani contributes to reduced virulence
Source: PLoS Negl Trop Dis. 2021 Feb 23;15(2):e0009079. doi: 10.1371/journal.pntd.0009079 (PMC7901767; doi:10.1371/journal.pntd.0009079)
Supplement: S1 Table — Comparison of the proportion of metacyclic cells present in stationary phase cultures. (PDF) [file pntd.0009079.s001.pdf]

S1 Table. A table showing the percentages of metacyclic like promastigotes present in the stationary phase cultures of wild type *L. donovani*, R231C RagC mutant and the RagC null mutant.

	Cell density in stationary phase	Metacyclic cells from 2 x 10^8 stationary cells	The metacyclic promastigotes rate (%)
WT *L. donovani*	7.925 x 10^7/ml	1.475 x 10^6	0.7375
R231C RagC	6.325 x 10^7/ml	7.4 x 10^6	3.7
RagC null mutant	6.425 x 10^7/ml	6.71 x 10^6	3.355
