# Supplementary figures and images for: Evidence that a naturally occurring single nucleotide polymorphism in the RagC gene of Leishmania donovani contributes to reduced virulence
Source: PLoS Negl Trop Dis. 2021 Feb 23;15(2):e0009079. doi: 10.1371/journal.pntd.0009079 (PMC7901767; doi:10.1371/journal.pntd.0009079)

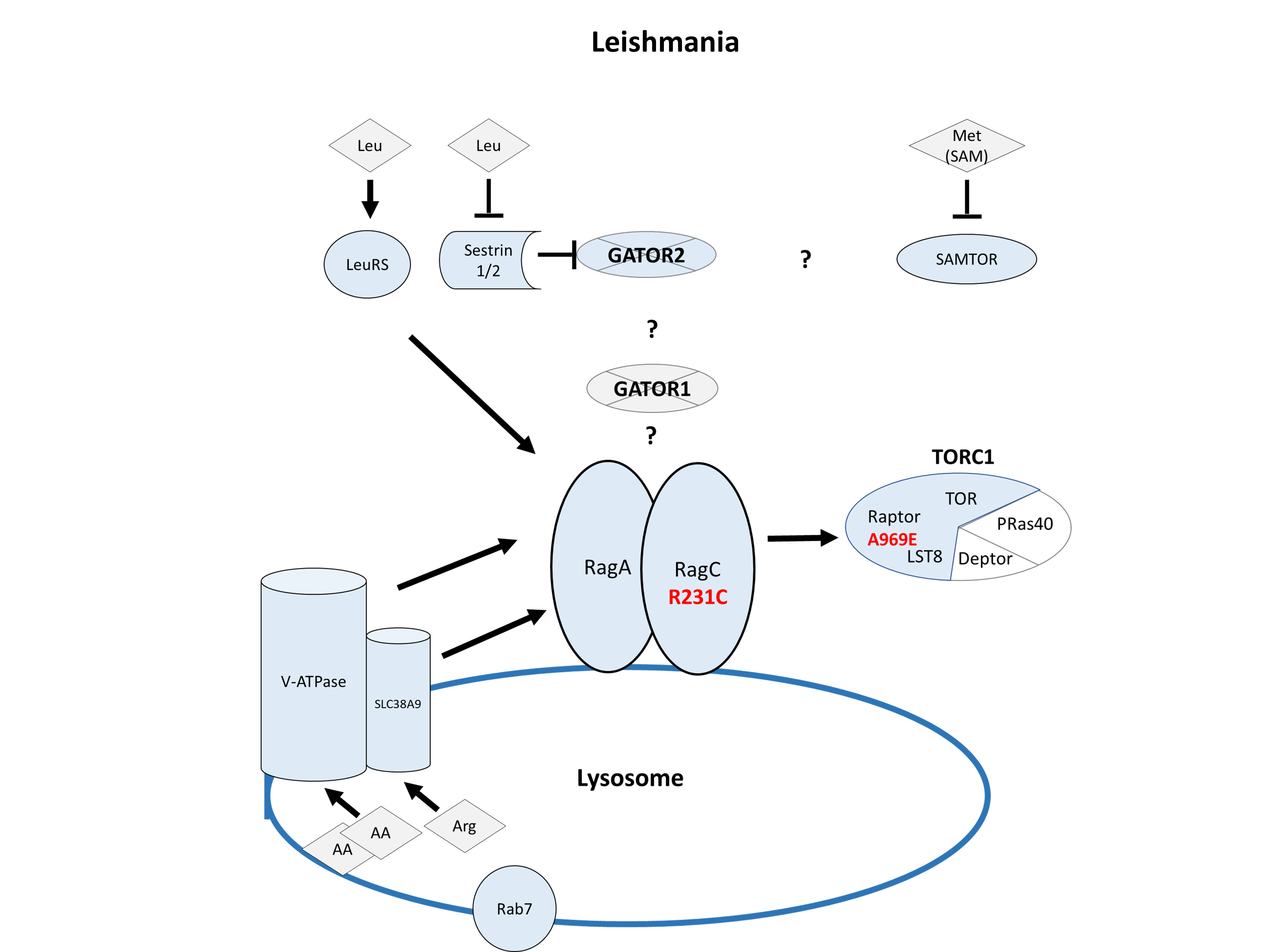

Supplement: S1 Fig — Components of the Rag pathway conserved in Leishmania based on sequence homology determined through bioinformatic analysis of the Leishmania genome. Note, there are several human components that were not identified in Leishmania including RagB and RagD. GATOR1 proteins were identified but with low homology or only partial member homology and are shown in grey. Also indicated are the RagC R231C, and Raptor A969E mutants in red. (TIF) [file pntd.0009079.s002.tif]

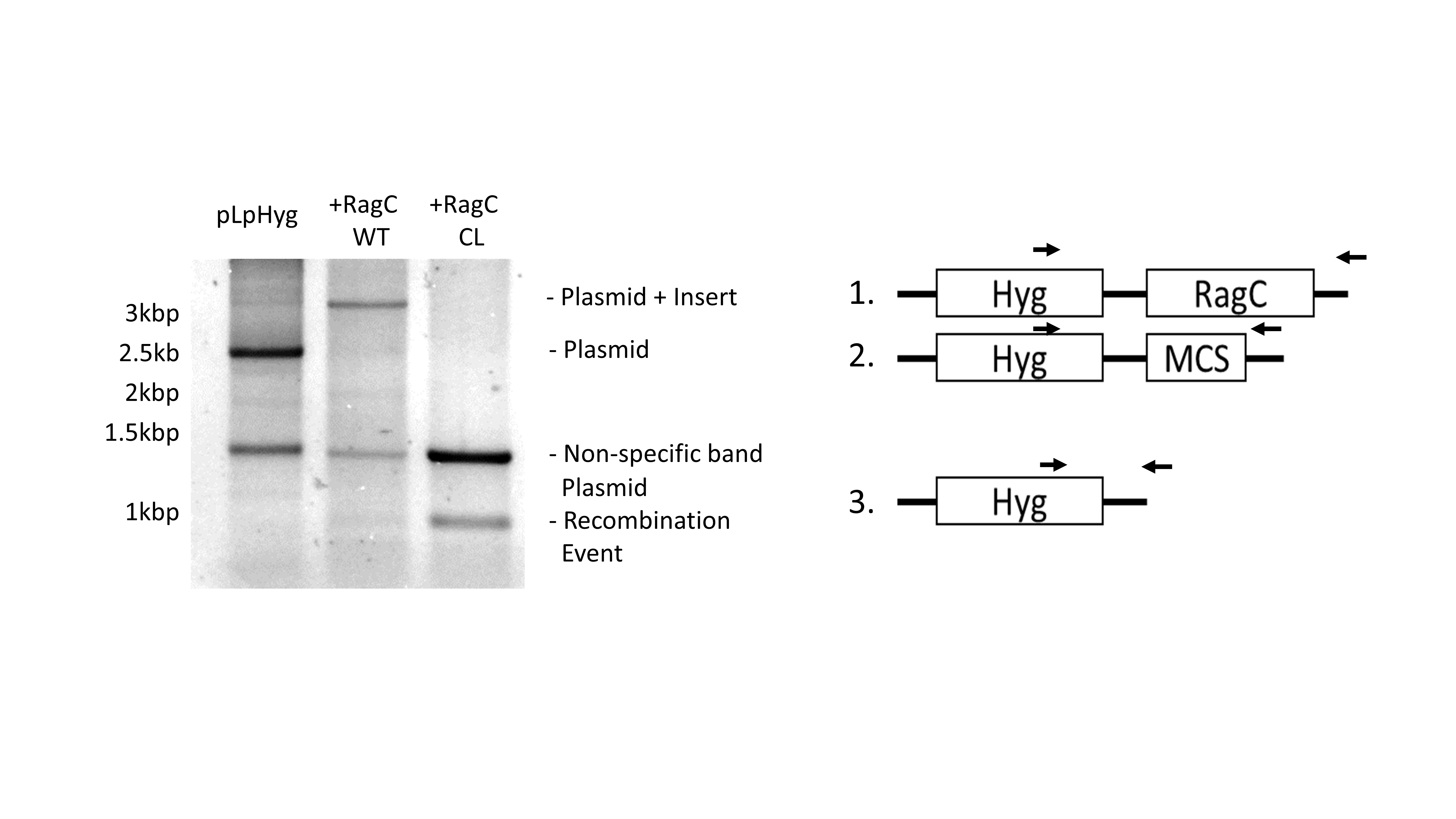

Supplement: S2 Fig — WT promastigotes were transfected with plasmids encoding either the WT or mutant copy of RagC. At 4 weeks post transfection the plasmids were recovered using a plasmid DNA mini-preparation kit. Primers (arrows) were designed to the C terminal end of the hygromycin resistance cassette (Hyg) and downstream of the protein insertion site (MCS) in order to target episomal copies of RagC only. The first lane products originate from an empty plasmid (Band type 2). The second lane shows an increase in band size due to the insertion and retention of the RagC protein on the plasmid (Band type 1). The third lane shows the excision of not only the RagC protein but the flanking UTR as indicated by the large decrease in size of amplicon (Band type 3). (TIF) [file pntd.0009079.s003.tif]

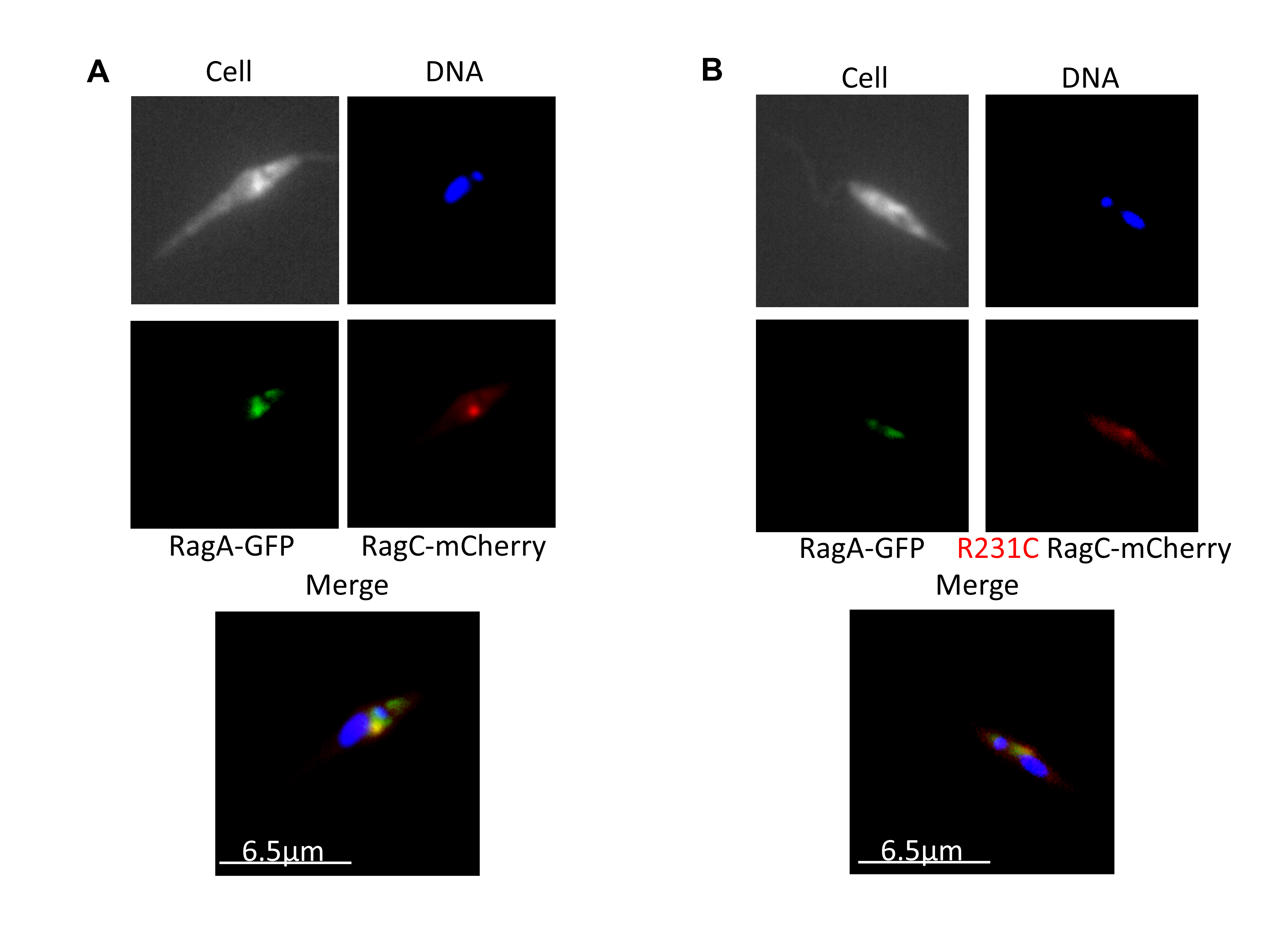

Supplement: S3 Fig — A. Epifluorescence microscopy of RagA and RagC. GFP-tagged RagA and mCherry-tagged RagC form overlapping foci near the kinetoplast and nucleus in transfected promastigotes. B. Epifluorescence microscopy of RagA and RagC R231C. GFP-tagged RagA and mCherry-tagged RagC R231C mutant form overlapping foci near the kinetoplast and nucleus in transfected promastigotes. (TIF) [file pntd.0009079.s004.tif]

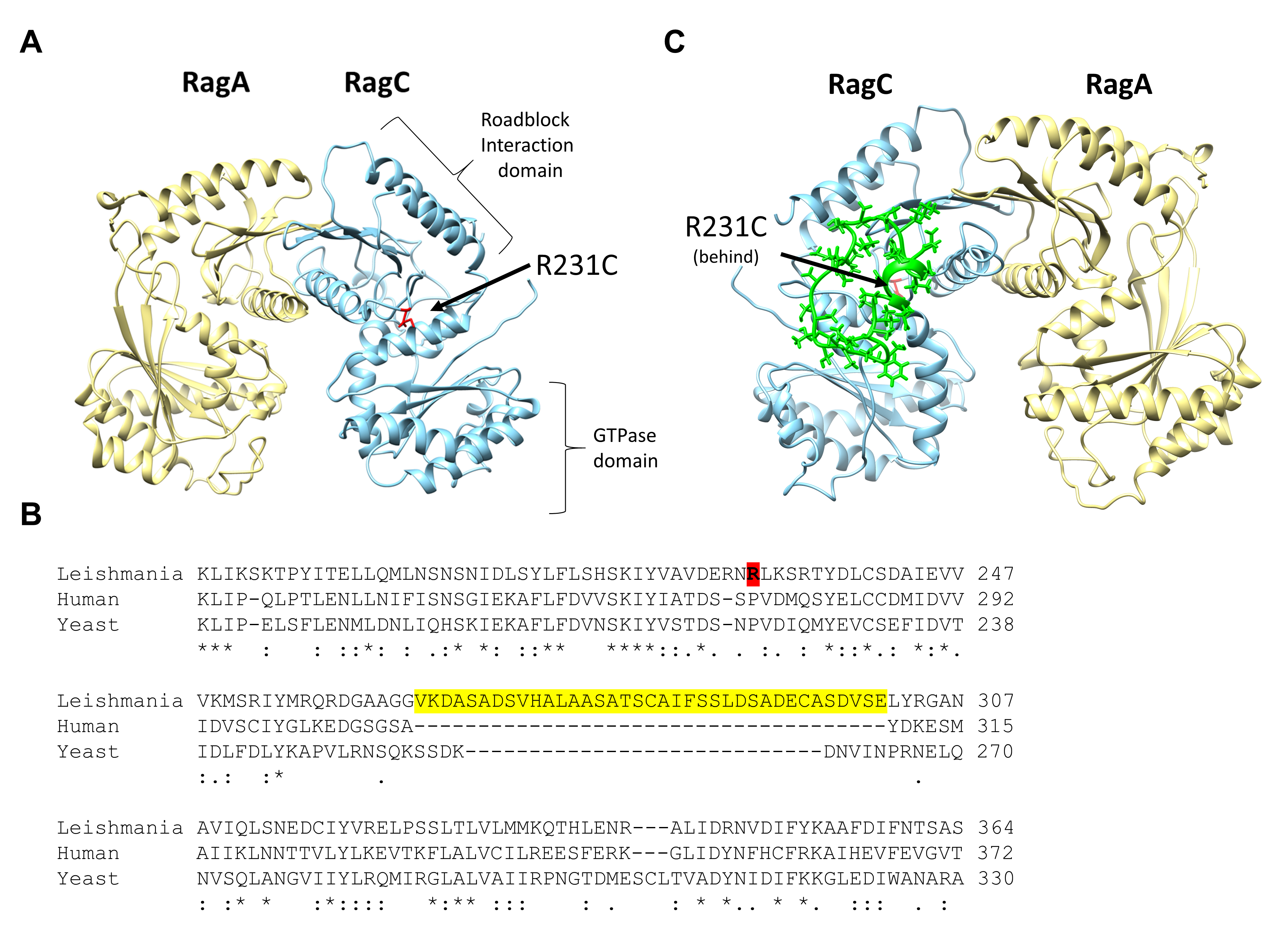

Supplement: S4 Fig — A. Homology modelling of Leishmania donovani RagA (yellow) in complex with RagC R231C (blue) shown in ribbon representation. The 231st amino acid position on RagC corresponding to the identified mutation is highlighted in red full atom representation and indicated by arrows. The complex is oriented with the Roadblock interaction domains shown on top and the GTPase domains at the bottom. B. Multiple sequence alignment between Leishmania, human and yeast RagC homologue sequences with L. donovani RagC R231 highlighted. C-terminal portion of the alignment shown with a 37 amino acid long sequence only seen in Leishmania highlighted C. Homology model of the RagA/RagC complex with the Leishmania specific sequence from B. shown in green. (TIF) [file pntd.0009079.s005.tif]

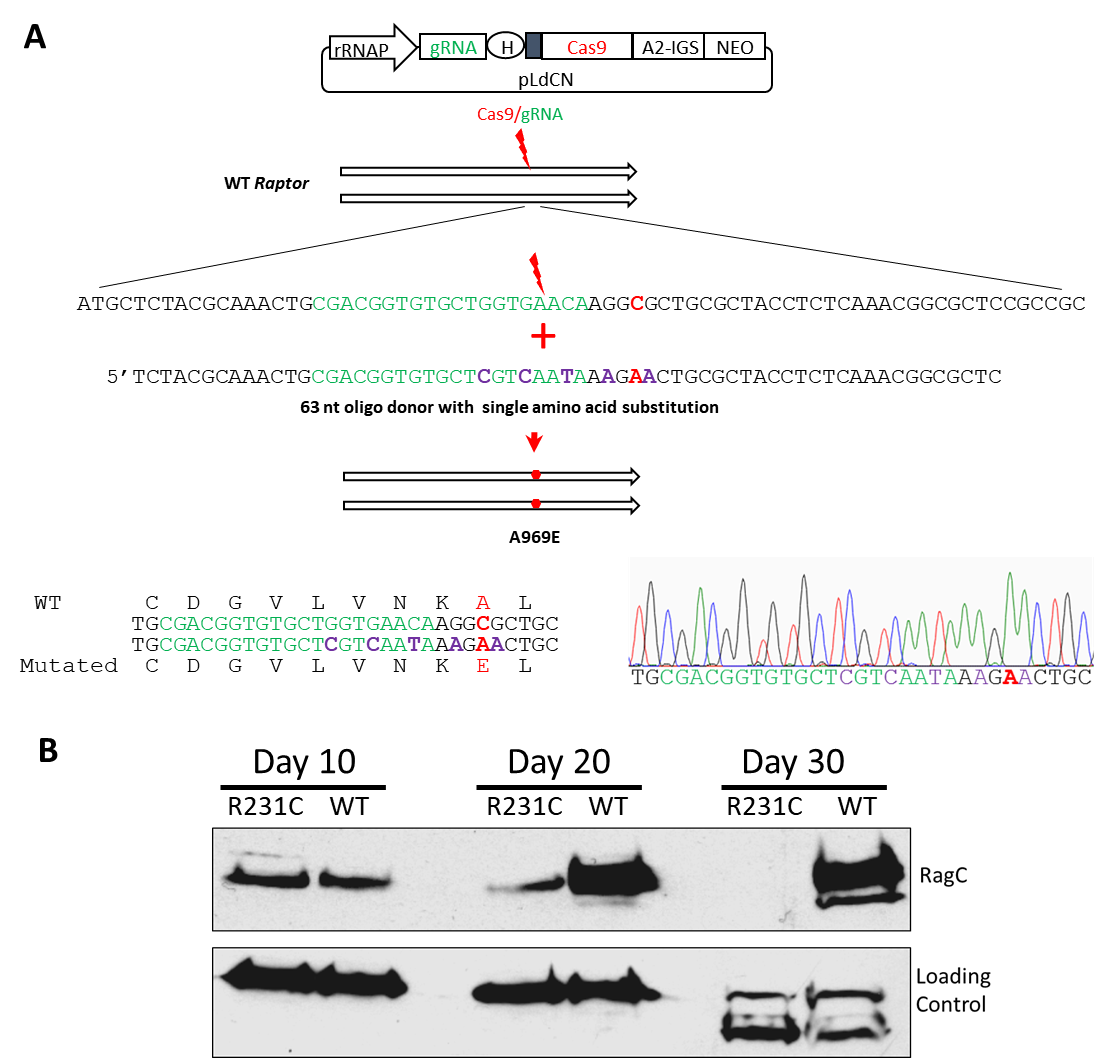

Supplement: S5 Fig — A. Strategy used to insert the Raptor A969E mutation into the Raptor gene of L. donovani. A gRNA was designed to target the Raptor gene at the site of the desired C to A base change and cloned into the CRISPR vector (pLdCNld251140) expressing this gRNA and Cas9 nuclease. This plasmid was transfected into 1S2D L. donovani cells followed by the transfection of a 63 nt donor oligonucleotide with 25 nt flanking sequences to introduce the desired C to A conversion (red) and 5 silent mutations to prevent further cleavage by Cas9 (purple). The cells were then cloned into 96-well plates and screened by PCR and Sanger sequencing. B. Immunoblot following the expression of RagC isoforms in the Raptor mutant parasites. The isolated parasites were transfected with plasmids expressing either the WT or R231C isoforms of RagC. At 10 days post-transfection, both isoforms are expressed at comparable low levels. At 20 days, the expression of the WT isoform is stabilized, the R231C isoform appears at slightly reduced levels compared to the 10-day time point. At 30 days post transfection, only the WT isoform is stably expressed. (PNG) [file pntd.0009079.s006.png]
